# Links Between Co-occurring Social-Communication and Hyperactive-Inattentive Trait Trajectories

**DOI:** 10.1016/j.jaac.2011.05.015

**Published:** 2011-09

**Authors:** Beate St. Pourcain, William P. Mandy, Jon Heron, Jean Golding, George Davey Smith, David H. Skuse

**Affiliations:** a Medical Research Council Centre for Causal Analysis in Translational Epidemiology, School of Social and Community Medicine, University of Bristol; b School of Social and Community Medicine, University of Bristol; c Institute of Child Health, University College London

**Keywords:** social-communication trait, hyperactive-inattentive trait, maternal smoking, teenage pregnancy, ALSPAC

## Abstract

**Objective:**

There is overlap between an autistic and hyperactive-inattentive symptomatology when studied cross-sectionally. This study is the first to examine the longitudinal pattern of association between social-communication deficits and hyperactive-inattentive symptoms in the general population, from childhood through adolescence. We explored the interrelationship between trajectories of co-occurring symptoms, and sought evidence for shared prenatal/perinatal risk factors.

**Method:**

Study participants were 5,383 singletons of white ethnicity from the Avon Longitudinal Study of Parents and Children (ALSPAC). Multiple measurements of hyperactive-inattentive traits (Strengths and Difficulties Questionnaire) and autistic social-communication impairment (Social Communication Disorder Checklist) were obtained between 4 and 17 years. Both traits and their trajectories were modeled in parallel using latent class growth analysis (LCGA). Trajectory membership was subsequently investigated with respect to prenatal/perinatal risk factors.

**Results:**

LCGA analysis revealed two distinct social-communication trajectories (persistently impaired versus low-risk) and four hyperactive-inattentive trait trajectories (persistently impaired, intermediate, childhood-limited and low-risk). Autistic symptoms were more stable than those of attention-deficit/hyperactivity disorder (ADHD) behaviors, which showed greater variability. Trajectories for both traits were strongly but not reciprocally interlinked, such that the majority of children with a persistent hyperactive-inattentive symptomatology also showed persistent social-communication deficits but not vice versa. Shared predictors, especially for trajectories of persistent impairment, were maternal smoking during the first trimester, which included familial effects, and a teenage pregnancy.

**Conclusions:**

Our longitudinal study reveals that a complex relationship exists between social-communication and hyperactive-inattentive traits. Patterns of association change over time, with corresponding implications for removing exclusivity criteria for ASD and ADHD, as proposed for *DSM-5*.

The expression of autism spectrum disorder (ASD) traits and attention-deficit/hyperactivity disorder (ADHD) traits in children from the general population is nonindependent.^1-3^ This is consistent with the high levels of comorbidity observed between ASD and ADHD, each of which is presumed to lie at the upper extreme of an underlying behavioral continuum.^4,5^ Children with ASD or pervasive developmental disorder (PDD) often have ADHD symptoms;^6-11^ recent reports indicate that 31% of children and adolescents with autism,^6^ 45% with PDD–Not Otherwise Specified (NOS),^7^ and 28% to 53% with ASD^8,11^ meet ADHD criteria as outlined in the *Diagnostic and Statistical Manual of Mental Disorders—4th edition* (*DSM-IV*).^12^ Conversely, autistic symptoms are often found in children with ADHD,^9,13,14^ especially social interaction and communication impairments,^9,13,14^ but also repetitive behaviors.^9^ This phenotypic overlap is supported by family and twin research, which produced evidence for shared genetic influences between autistic and ADHD related symptoms,^15^ both throughout normal population variation^1-3^ and at the extreme.^16,17^

Little is known however, as to how the relationship between autistic and ADHD symptoms changes over time. Autistic traits^18,19^ and ASD diagnoses^20^ are in general persistent during the course of development, with *DSM-IV*–based diagnostic stability estimates ranging from 69 to 95%.^21,22^ In contrast, ADHD diagnoses^23^ and the expression of ADHD symptoms^24^ are more variable. There is some evidence for stability of ADHD diagnoses across time^23,24^ (*DSM-IV*–based diagnostic stability estimates, 15%–65%), especially for the combined hyperactive-impulsive/inattentive ADHD subtype.^25^ However, ADHD symptoms may decline with age,^23,25^ but may also increase during adolescence,^24,26^ suggesting heterogeneity in the underlying ADHD trajectories. The relationship between autistic and ADHD related symptoms may therefore vary over time, and some ADHD related trajectories might be more strongly related to autistic symptoms than others. Moreover, it is possible that, depending on this interrelationship, risk factors for both symptomatologies may indeed be shared.

Although genetic effects are strongly implicated in the co-development of ASD and ADHD traits, they do not account for all of their phenotypic covariation,^1-3^ implying that environmental risk factors could be common to both conditions. Support for this latter hypothesis comes from several epidemiological studies, which suggested the existence of overlapping prenatal/perinatal influences. Maternal immune activation by infections and maternal substance use during pregnancy, in particular maternal smoking, have been suggested as risk factors for both ADHD^27-29^ and autism.^30,31^ In addition, perinatal complications (such as being born prematurely or having a lower birth weight) may play a role in the development of both ADHD-related^27,32^ and autistic symptoms.^33,34^ Some research has also linked maternal age at birth to both symptomatologies, although it may reveal trait-specific risk patterns, as an advanced maternal age has been associated with autism^33^ and a younger maternal age with ADHD.^32^ Examining the influence of these risk factors upon jointly modeled autistic and hyperactive symptom trajectories with a view to identifying the antecedents of co-occurring symptoms will therefore facilitate the identification of common etiologies.

Using the Avon Longitudinal Study of Parents and Children (ALSPAC), a longitudinal population-based birth cohort, this work explored the developmental trajectories of autistic and ADHD traits in a general population sample. The selection of autistic symptoms focussed exclusively on the social-communication spectrum of ASD, which is likely to be etiologically distinct from the repetitive behavioral spectrum.^35^ Investigated ADHD symptoms comprised the entire ADHD triad, including symptoms of inattention, hyperactivity, and impulsivity.^36^ In the presented work, we a) interrogated the interrelationship between co-occurring social-communication and hyperactive-inattentive trait trajectories to explore links between trait-specific trajectory types; and b) investigated the evidence for shared prenatal/perinatal risk factors, in particular those that have been previously related to both social-communication and hyperactive-inattentive symptoms on an individual trait basis.

## Method

### Study Samples

ALSPAC is a population-based, longitudinal, pregnancy-ascertained birth cohort in southwest England with an estimated date of birth between April 1, 1991, and December 31, 1992;^37^ the initial cohort included 14,541 pregnancies and 13,971 children were alive at 12 months of age (>95% of white European origin). A detailed description of the cohort has been published previously.^37^ Ethical approval was obtained from the ALSPAC Law and Ethics Committee and the Local Research Ethics Committees. Eligible children for this analysis were white European singletons (12,627 of 13,971 children) with a total intelligence quotient (IQ) of ≥70 at 8 years of age (6,536 of 12,627 children had available intelligence scores, of which 6,418 were eligible). Children excluded because of lower cognitive functioning showed increased rates of both social-communication deficits (22%–31% between 8 and 17 years) and hyperactive-inattentive symptoms (18%–42% between 4 and 17 years, as defined below), and may share a common etiology primarily because of deficits in cognitive resources. Overall, ineligible white singleton children were more likely to have been born to mothers who had the following characteristics, when compared with eligible children: adolescent (<20 years; odds ratio [OR] 4.65, 95% confidence interval [95% CI] = 3.76–5.81); average (OR = 2.31, 95% CI = 2.12–2.50) or less than average education (OR = 2.31, 95% CI = 2.02–2.64), or in manual occupations (OR = 2.26, 95% CI = 2.05–2.50). As such ALSPAC, like other cohort studies, is prone to selective dropout, in particular with respect to socio-economic position. This may lead to an underestimation of the prevalence of a developmental trajectory, and will have an impact on power.^38^ However, a recent empirical study and simulations on the ALSPAC sample showed that this selective dropout only marginally affects regression models with respect to behavioral outcomes.^38^

Attrition rates among the 6,418 eligible children varied between 14.0% and 35.9% for social communication scores at 8 to 17 years of age, and between 11.2% and 36.1% for hyperactive-inattentive scores at 4 to 17 years of age. To further facilitate the identification of growth trajectories and the convergence of the complex statistical models (discussed below), all eligible individuals with more than 50% missing data for either social-communication or hyperactive-inattentive symptom scores were excluded, resulting in a total sample of 5,383 individuals (2,669 male and 2,714 female participants).

### Measurement of Prenatal Risk Factors

The search for shared prenatal risk factors focused on the first trimester, as especially during this time-window risk factors for both autistic symptoms,^30,31^ reported during the earlier stages of pregnancy, and ADHD symptoms,^27-29^ reported throughout pregnancy, may overlap. Information on maternal substance use with respect to alcohol, tobacco (cigarette smoking) and cannabis use, influenza-like illnesses, and any infections was ascertained with questionnaires at 18 weeks of gestation (Supplement 1, available online).

Given the possibility that the effect of maternal smoking may manifest through familial influences,^39,40^ we also investigated the association with paternal smoking^41^ as part of a sensitivity analysis. Information on paternal (cigarette) smoking during early pregnancy was obtained with questionnaires at 18 weeks of gestation (Supplement 2, available online).

### Measurement of Perinatal Information

Data on low birth weight, preterm birth, and maternal age at birth were collected at birth, and information on parity was ascertained with questionnaires at 18 weeks of gestation (Supplement 3, available online).

### Measurement of Socio-Economic Position

Information on occupational social class and maternal education was obtained using questionnaires at 32 weeks of gestation (Supplement 4, available online).

### Measurement of Social-Communication Traits

Social-communication skills were captured with the 12-item Social Communication Disorder Checklist (SCDC^42^; score range 0–24; Supplement 5, available online). The SCDC is a brief screening instrument of social reciprocity and verbal/nonverbal communication^42^ (age range 3–18 years) that has high sensitivity and specificity for autism^43^ with higher scores reflecting more social-communication deficits. Mother-reported SCDC scores for children and adolescents were assessed at 8, 11, 14, and 17 years of age, and all scores showed a high temporal stability (0.38<rho<0.58; Table S1, available online). For the presented work, high-scoring individuals were identified based on a cut-off at ≥9 scores, which has been previously shown to provide maximum discrimination between all PDD diagnoses and non-PDD diagnoses/normal comparisons.^42^

### Measurement of Hyperactive-Inattentive Traits

The Strengths and Difficulties Questionnaire (SDQ)^36^ is a behavioral screening questionnaire (age range 4–16 years) with high reliability and validity with respect to the identification of a psychiatric diagnosis.^44^ The questionnaire assesses hyperactivity-inattention with a five-item subscale (score range 0–10), with higher scores indicating more behavioral problems (three hyperactive-impulsive and two inattentive items; Supplement 6, available online). Mother reports on their children's hyperactivity-inattention were obtained at 4, 7, 8, 10, 12, 13, and 17 years of age, and there was a high temporal stability among all symptom scores (0.34<rho<0.71; Table S2, available online). Pertinent to this analysis, high-scorers were identified using a cut-off at ≥7 scores, which is indicative of abnormal behavior.^36^

Sample characteristics for all outcome measurements, potential confounders and risk factors are given in Table S3, available online.

### Statistical Analysis

The statistical analysis was carried out within two parts. Within the first part, trajectories of social-communication deficits and hyperactive-inattentive symptoms were modeled in parallel using latent class growth analysis (LCGA) as implemented within MPlus, v.6.1 (Muthén and Muthén, Los Angeles, CA). Similar to a growth mixture model (GMM), LCGA aims to identify a multinomial latent class variable, which corresponds to different underlying growth curve shapes of child behavior measured across multiple time points.^45^ In contrast to GMMs, however, all elements of the within-class covariance matrix of the growth factors are constrained to zero,^45^ and, as such, LCGA does not rely on the within-class normality assumption of random effects. LCGA allows the trajectory modeling of parallel outcomes with multiple measurements, including up to 7 repeated measures per trait as proposed within this study, which is computationally not yet feasible with GMM using dual-core Windows processors.

**FIGURE 1:**
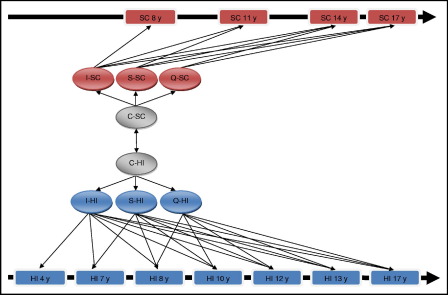
Parallel latent class growth analysis model structure for social-communication and hyperactive-inattentive traits. Note: C = latent trajectory classes; HI = hyperactive-inattentive symptoms; I = intercept growth parameter; Q = quadratic growth parameter; S = slope growth parameter; SC = social-communication deficits; y = years (children's age at measurement).

Using a parallel LCGA approach, trajectories for both social-communication and hyperactive-inattentive traits were each modeled using intercept, slope, and quadratic growth parameters. Specifically, a series of parallel LCGA models was fitted as a combination of one to three class models for social-communication traits and one to five class models (more classes did not provide stable model estimates) for hyperactive-inattentive traits. A graphical representation of the overall model structure is given in Figure 1. Missing data were accounted for through full information maximum likelihood. All models with two or more trajectories per trait were allowing for correlations between cross-trait trajectories through a log-linear link. As such social-communication trait trajectories and hyperactive-inattentive trait trajectories were modeled conditionally dependent on each other with the modeling of shared class membership being part of the same LCGA model. Posterior probabilities for combinations of social-communication and hyperactive-inattentive trait trajectories were based on joint posterior probabilities (i.e., the posterior probability of social-communication trait trajectories × the posterior probability of hyperactive-inattentive trait trajectories). The improvement in model fit was captured through the Bayesian information criterion (BIC), with lower BIC indicating a more parsimonious model fit. In addition, the model selection was guided through the evaluation of the model classification accuracy, with MPlus entropy values closer to 1 (range 0–1) indicating a higher precision. Sex-specific differences in developmental outcomes were captured as differences in trajectory proportions, i.e., including sex as a covariate in the regression equation. By contrast, models with sex-specific trajectories (allowing for sex-specific growth parameters) did not converge. Sex-specific analysis was eventually integrated within the regression models outlined below.

Within the second part of the analysis, we used logistic and multinomial regression frameworks in STATA 11.1 (Stata Corporation, College Station, TX) to investigate the influence of potential risk factors on jointly modeled social-communication and hyperactive-inattentive trait trajectories respectively, using the best-fitting model from part 1. As MPlus-derived trajectory memberships are probabilistic, we generated 250 datasets in which assignments of trajectory classes for each individual were based on random draws from the distribution of joint posterior probabilities. The effect of each risk factor was studied using an individual regression model and adjusted for sex and potential confounders (i.e., social class and maternal education). Presented estimates were combined for each predictor across all datasets using the STATA *mi* command.

## Results

### Developmental Trajectories of Social-Communication and Hyperactive-Inattentive Traits

**FIGURE 2:**
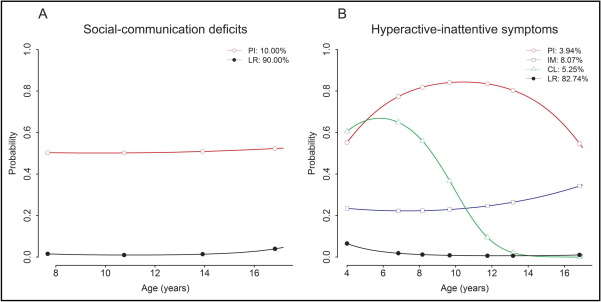
Trajectories of social-communication deficits (A) and hyperactive-inattentive symptoms (B). Note: Each trait trajectory shows the probability of expressing social-communication deficits (A) or hyperactive-inattentive symptoms (B), with respect to the selected cut-off for high-scoring individuals. CL = childhood-limited; IM = intermediate; LR = low-risk; PI = persistently impaired.

Parallel LCGA modeling showed that the most parsimonious model with the best BIC fit in combination with a high classification accuracy comprised two social-communication trait trajectories and four hyperactive-inattentive trait trajectories (Table S4, available online). Latent class trajectories for social-communication traits identified a persistently impaired group (10.00%) with a high probability of expressing deficits in social reciprocity and verbal/nonverbal communication throughout development, and a low-risk group (90.00%) (Figure 2A). ADHD-related developmental pathways during childhood and adolescence were described by four distinct trajectory classes (Figure 2B): 1) persistently impaired children with a high probability (probability >0.5) of expressing hyperactive-inattentive symptoms (3.94%); 2) children with an intermediate probability (0.2 < probability < 0.4) of expressing these symptoms (8.07%); 3) a group of children with a childhood-limited expression pattern of hyperactive-inattentive symptoms (5.25%); and 4) a low-risk group (82.75%). Joint probabilities for social-communication and hyperactive-inattentive trait trajectories revealed the tight interrelationship between both traits (Table 1). This link was strongest between the two persistently impaired groups, with respect to both magnitude and strength of the association (log-linear estimates and z-scores are detailed in Table 1). Most children of the persistently impaired social-communication group were either part of the persistently impaired hyperactive-inattentive group (32.29%) or the intermediate hyperactive-inattentive group (39.01%). Only a few of them fell into the childhood-limited hyperactive-inattentive (12.14%) or low-risk hyperactive-inattentive categories (16.56%). Children with a persistently high probability of hyperactive-inattentive symptoms by contrast were almost entirely included within the persistently impaired social communication group (82.04%). In addition, 48.30% children of the intermediate hyperactive-inattentive group, 23.09% children of the childhood-limited hyperactive-inattentive group, and 2.00% children of the low-risk hyperactive-inattentive group had an increased probability for persistent social-communication deficits (conditional probabilities are based on the log-linear estimates in Table 1). An adjustment for sex did not affect the observed trait interrelationships (Table S5, available online).

### Predictors of Social-Communication and Hyperactive-Inattentive Trait Trajectories

The strongest predictor for both social-communication deficits and hyperactive-inattentive behavior was male sex (Table 2). This can be translated into sex-specific trajectory proportions: For hyperactive-inattentive trajectories, 5.92% of male participants were in the persistently impaired group, 9.64% in the intermediate group, 6.52% in the childhood-limited group, and 77.92% in the low-risk group. The proportions of these trajectories in females were 1.94%, 6.51%, 4.01%, and 87.54%, respectively. Furthermore, 12.74% of male participants versus 7.20% of female participants had persistently impaired social communication skills, whereas 87.26% of males versus 92.80% of females belonged to the social communication low-risk group. The strongest socio-economic predictor for both social-communication deficits and hyperactive-inattentive symptoms, especially for persistently impaired children, was a lower level of maternal education (Table 2).

Shared predictors for both traits after accounting for the influences of sex and socio-economic position were maternal cigarette smoking during the first trimester and a teenage pregnancy (Table 2). For each predictor, the strongest relationships were observed with persistent social-communication deficits and persistent hyperactive-inattentive symptoms, respectively. Both risk factors are likely to act independently as the association was only marginally attenuated when their effects were controlled for each other (data not shown). Sensitivity analysis showed, however, that an adjustment for early paternal smoking during pregnancy^41^ weakened the association for maternal smoking with respect to both traits (Table 3), although a maternal risk effect for persistently impaired hyperactive-inattentive behavior was still present. On the other hand, there was no evidence for an association between paternal smoking and persistent behavioral problems independently of maternal smoking effects. To further characterize the influence of a teenage pregnancy with respect to social-communication deficits, children of the persistently impaired group were divided into individuals with and without co-occurring persistent hyperactive-inattentive behavior. This sensitivity analysis revealed that an adolescent pregnancy was a risk factor only for children with persistent social-communication deficits *and* persistent hyperactive-inattentive symptoms (OR = 4.57, 95% CI = 1.68–12.39; *p* = .003), but not for children with persistently impaired social-communication skills without a persistent hyperactive-inattentive symptomatology, i.e., in combination with an intermediate, childhood-limited, or low-risk hyperactive-inattentive profile (OR = 1.17, 95% CI = 0.29–4.68; *p* = .82; reference group: social-communication low-risk; data not shown).

In addition, we observed a trend for an association between infections during the first trimester and persistent social communication deficits only (Table 2).

## Discussion

Adopting a longitudinal perspective, this study observed strong links between co-occurring social-communication and hyperactive-inattentive traits in a general population sample. This developmental finding corroborates previous cross-sectional research.^1-3,6-11,13-17^ More importantly however, our findings reveal a novel temporal insight into the complexity of this trait interrelationship through the identification and association analysis of trait-specific developmental pathways. In particular, our longitudinal approach identified two social-communication domain related autistic trait trajectories (persistently impaired versus low-risk) and four hyperactive-inattentive trait trajectories (persistently impaired, intermediate, childhood-limited, and low-risk). This is consistent with the reported higher stability of autistic symptoms^18-22^ and the greater variability of ADHD-like behavior^23,24,26^ during the course of child development. Among the hyperactive-inattentive trajectories, the persistently impaired and childhood-limited groups each matched previous reports on stable ADHD symptoms^23-25^ and the decline of ADHD symptoms with progressing age,^23,25^ respectively. The hyperactive-inattentive intermediate trajectory with a trend for an increased probability of expressing symptoms during adolescence may also correspond to existing observations.^24,26^ Above all however, our study reported for the first time that the observed trait interrelationship between the most persistently impaired individuals is not reciprocal. Although the majority of children with persistently impaired social-communication skills were either part of the persistently impaired hyperactive-inattentive *or* the intermediate hyperactive-inattentive group, children with persistent hyperactive-inattentive symptoms were almost entirely subsumed within the persistently impaired social-communication group. In other words, almost all children who exhibited a high probability for persistent hyperactive-inattentive symptoms had also a high probability for persistent social communication deficits, but not vice versa. Interestingly, the ADHD combined subtype, based on both a DSM-IV diagnosis and population-derived latent classes, also had the highest stability across time^25^ and the highest autistic symptom scores^46^ in previous research.

The current *DSM-IV-TR*^12^ prohibits a diagnosis of ADHD when the symptoms occur during the course of a PDD, based on the rationale that these ADHD-related symptoms are primarily attributable to the autistic disorder. However, the strong trajectory links observed in our study, especially between the most persistently affected individuals, directly support the proposed revisions for the new *DSM-5.* These include changes with respect to the diagnostic ADHD criteria with ASD comorbidity, which will allow ASD and ADHD to be diagnosed in the same individual (http://www.dsm5.org/ProposedRevisions/). Our results may therefore be of clinical significance with respect to the refinement of the phenotypic ASD/ADHD overlap from a longitudinal perspective, and may indeed reflect the existence of a novel autistic/hyperactive-inattentive syndrome. This hypothesis finds general support through genetic analyses,^15^ including both general population traits^1-3^ and genetic studies of individuals with severe symptoms.^16,17^ Furthermore, several common genetic variants, such as recently identified ADHD and ASD genome-wide analysis signals,^15^ may have relevance for both conditions. Our findings are also consistent with the identification of clinical^47^ and distinct genetic^17^ subtypes of ADHD, with and without autistic symptoms. Latter may correspond to the observed weaker overlap between hyperactive-inattentive childhood-limited trajectories and persistently impaired social-communication skills. It is furthermore possible that children with a combined persistently impaired social-communication and hyperactive-inattentive phenotype may become more prominent clinically during the course of development, as other developmental pathways diverge. Thus, the hypothesis of a novel persistent autistic/hyperactive-inattentive phenotype could explain the previously reported increase in genetic correlation between autistic and ADHD traits with progressing age,^3^ rising from 0.23 to 0.26 in 2-year old children,^3^ to 0.54 to 0.57 during middle childhood^2^ to 0.72 in early adulthood.^1^

Finally, we also considered the hypothesis that the correlation between social-communication and hyperactive symptom trajectories could imply, beside genetic-factors, a nongenetic etiology. This is supported by our findings of shared prenatal/perinatal predictors for social-communication *and* hyperactive-inattentive trait trajectories, which included maternal tobacco smoking during the first trimester and a teenage pregnancy. These effects were most closely linked to the persistently impaired social-communication and persistently impaired hyperactive-inattentive trajectories.

Maternal smoking during pregnancy is a commonly reported predictor for both autistic and ADHD-related symptoms.^27,29,30^ In line with previous studies however, our results implied that the observed effect could to a considerable extent reflect familial influences,^39,40^ as we observed similar links between maternal and paternal smoking with respect to persistent behavioral problems (although we cannot exclude the possibility of passive smoking effects). Familial smoking effects may manifest as shared unaccounted social environmental but also as genetic factors,^39-41^ especially as paternal smoking has been suggested as a proxy for ADHD and/or smoking risk genes.^48^ On the other hand, maternal smoking was associated with persistent hyperactive-inattentive behavior independent of paternal smoking, and it is thus possible that some aspects of maternal smoking also influence as exposure in utero mental health across the life course.

The observed risk effects associated with a teenage pregnancy are compatible with previous reports linking younger maternal age and an ADHD symptomatology,^32^ but contrast with autism-associated risk and advanced maternal age at birth.^33^ Closer examination showed, however, that a teenage pregnancy was predominantly associated with persistent social-communication deficits in combination with persistent hyperactive-inattentive symptoms, but not without a persistent hyperactive-inattentive symptomatology.

In line with the observed trajectory interrelationships, the identification of shared predictors highlights therefore the possibility that a common comorbidity between social-communication and hyperactive-inattentive traits might only be inherent to specific trajectory combinations. On the other hand, children may vary in combinations of continuously distributed phenomena without the presence of specific endophenotypes. Testing the hypothesis of an underlying autistic/hyperactive-inattentive syndrome will require the adoption of a new research perspective, which understands social-communication deficits and hyperactive-inattentive symptoms not only as correlated single traits but as part of a joint phenotype. Modeling the developmental pathways of such a presumed combined syndrome by identifying classes of conditionally independent joint social-communication *and* hyperactive-inattentive trajectories will then facilitate the identification of endophenotypic characteristics.

These findings must be interpreted within the context of potential limitations. First, measures of social-communication traits, hyperactive-inattentive traits, and prenatal risk factors were predominantly based on mother-report and may have contributed to a greater variance sharing. Second, autistic trajectories were explored only with respect to the social-communication domain, and it is possible that the repetitive behavioral spectrum of ASD has different developmental outcomes and relationships with co-occurring hyperactive-inattentive symptom trajectories. Likewise, the pattern of association might differ for symptoms of the ADHD combined type, compared with the predominantly inattentive or predominantly hyperactive-impulsive type,^10^ thus setting new targets for future research into linked trajectories of ASD and ADHD domains. Third, some of the assessed social-communication deficits may reflect social impairments that are the consequence of hyperactive-inattentive behavior itself. Concurrent validity analysis of the SCDC, however, convincingly demonstrated strong differences in SCDC scores between ASD groups and clinical control groups, including children with ADHD.^42^ Fourth, although we focused on the analysis of social-communication deficit and hyperactive-inattentive symptom trajectories using validated and standardized psychological instruments, and although the observed trait links were supported by independent genetic and observational research, we cannot exclude the possibility that other trait combinations may show similar trajectory associations in relation to the ASD domain. Finally, social-communication traits have been assessed only from age 8 years onward, and it is possible that earlier measures would have contributed to greater trajectory variability.

In summary, our study provided evidence for a strong interrelationship between social-communication and hyperactive-inattentive traits and their developmental pathways in childhood and adolescence, especially for persistently impaired children.

## Figures and Tables

**TABLE S1:** Temporal Stability of Social-Communication Deficits

Age (y)	8	11	14	17
8	1.00	—	—	—
11	0.58	1.00	—	—
14	0.49	0.58	1.00	—
17	0.38	0.45	0.55	1.00

Note: Temporal stability of social-communication deficits was assessed using Spearman's rank correlation coefficient. The analysis was based on a sample of eligible individuals with complete information on social-communication deficits across all time points (N = 3,511).

**TABLE S2:** Temporal Stability of Hyperactive-Inattentive Symptoms

Age (y)	4	7	8	10	12	13	17
4	1.00	—	—	—	—	—	—
7	0.54	1.00	—	—	—	—	—
8	0.52	0.70	1.00	—	—	—	—
10	0.48	0.67	0.70	1.00	—	—	—
12	0.43	0.61	0.66	0.69	1.00	—	—
13	0.40	0.57	0.60	0.64	0.71	1.00	—
17	0.34	0.45	0.49	0.54	0.58	0.60	1.00

Note: Temporal stability of hyperactive-inattentive symptoms was assessed using Spearman's rank correlation coefficient. The analysis was based on a sample of eligible individuals with complete information on hyperactive-inattentive symptoms across all time points (N = 3,182).

**TABLE S3:** Sample Characteristics (max *N* = 5,383)

Trait	Males (*N* = 2,669)			Females (*N* = 2,714)		
n (%)	Age, y	Total	n (%)	Age, y	Total
Mean (SD)	Mean (SD)
Social-communication deficits (High-scorer)	220 (8.7)	7.6 (0.1)	2,535	110 (4.3)	7.6 (0.1)	2,567
	188 (7.5)	10.7 (0.1)	2,511	97 (3.8)	10.7 (0.1)	2,545
	177 (7.6)	13.9 (0.1)	2,341	122 (5.1)	13.9 (0.2)	2,394
	161 (8.2)	16.8 (0.4)	1,953	179 (8.5)	16.8 (0.4)	2,105
	388 (15.3)	4.0 (0.3)	2,532	261 (10.2)	4.0 (0.4)	2,554
Hyperactive-inattentive symptoms (High-scorer)	303 (12.1)	6.8 (0.1)	2,502	151 (6.0)	6.8 (0.1)	2,535
	345 (14.1)	8.2 (0.2)	2,454	164 (6.6)	8.2 (0.2)	2,492
	222 (8.8)	9.6 (0.1)	2,518	109 (4.2)	9.6 (0.1)	2,566
	213 (8.9)	11.7 (0.1)	2,391	85 (3.5)	11.7 (0.1)	2,453
	203 (8.8)	13.2 (0.2)	2,319	89 (3.8)	13.1 (0.2)	2,365
	141 (7.2)	16.8 (0.4)	1,946	73 (3.5)	16.8 (0.4)	2,103

Socio-Economic Position	n (%)		Total	n (%)		Total
Nonmanual work	2,244 (88.9)		2,525	2,264 (87.4)		2,591
Maternal education			2,627			2,669
< O-level	220 (8.4)			202 (7.5)		
O-level	1,199 (45.6)			1,211 (45.4)		
> O-level (R)	1,208 (46.0)			1,256 (47.1)		

Prenatal Maternal Risk Factors (1^st^ Trimester)	n (%)		Total	n (%)		Total
Alcohol use			2635			2677
≥1 glass/wk	>1128 (42.8)			>1168 (43.6)		
<1 glass/wk	1089 (41.3)			1116 (41.7)		
Never (R)	418 (15.9)			393 (14.7)		
Smoking	432 (16.3)		2645	409 (15.2)		2686
Marijuana use	42 (1.6)		2553	38 (1.5)		2582
Influenza	208 (8.3)		2507	238 (9.4)		2529
Infections	577 (23)		2507	595 (23.5)		2529

Perinatal Risk Factors	n (%)		Total	n (%)		Total
Low birth weight	>101 (3.8)		2639	>76 (2.8)		2677
Premature birth	140 (5.2)		2669	101 (3.7)		2714
Multiparous	1372 (52.4)		2619	1390 (52.5)		2646
Maternal age			2669			2714
<20 years	31 (1.2)			35 (1.3)		
20–35 years (R)	2253 (84.4)			2363 (87.1)		
>35 years	385 (14.4)			316 (11.6)		

Note: R = reference.

**TABLE S4:** Parallel Latent Class Growth Analysis Model Fit Indices

No. of Fitted Latent Class Trajectories(Social-Communication Traits, Hyperactive-Inattentive Traits)	LL	BIC	Entropy	No. of Parameters
1,1^a^	−1,3999	28,049.52	—	6
1,2	−12,236.5	24,576.07	0.904	12
1,3	−12,140	24,417.53	0.859	16
1,4	−12,094.8	24,361.43	0.866	20
1,5	−12075.9	24,358.07	0.657	24
2,1	−13,348.4	26,799.94	0.883	12
2,2	−11,250.6	22,647.22	0.902	17
2,3	−11,128.5	22,445.99	0.869	22
**2,4**	−**11,067**	**22,365.92**	**0.860**	**27**
2,5	−11,046.3	22,367.47	0.869	32
3,1	−13,325.1	26,787.73	0.904	16
3,2	−11,215.6	22,620.17	0.884	22
3,3	−11,080.6	22,401.7	0.809	28
3,4^b^	−11,003.8	22,299.73	0.847	34
3,5	NE	NE	NE	NE

Note: Some data not estimated (NE) because of unstable model estimates (nonpositive definite first-order derivative product matrix when using 250 random starts). The selected latent class growth analysis (LCGA) model is indicated in boldface. BIC = Bayesian information criterion; LL = log-likelihood.

a The 1,1 model is identical to a LCGA model with one class only.

b Note that the 3,4 model had a lower BIC but also less classification accuracy compared with the more parsimonious 2,4 model.

**TABLE S5:** Relationships Between Social-Communication and Hyperactive-Inattentive Trait Trajectories Adjusted for Sex

Log-linear Estimates β (SE)			
	Hyperactive-Inattentive Trait Trajectories		
	PI^a^^,^^b^	IM^a^^,^^b^	CL^a^^,^^b^
**Social-Communication Trait Trajectories**			
**PI**^a^^,^^b^	5.56 (0.40) (z = 13.98)	3.81 (0.0.35) (z = 11.05)	2.81 (0.45) (z = 6.18)

Joint Class Probabilities (%)^c^					
	Hyperactive-Inattentive Trait Trajectories				
	PI	IM	CL	LR	Total
**Social-Communication Trait Trajectories**					
**PI**	3.26	3.92	1.27	1.55	10.00
**LR**	0.70	4.66	4.13	80.51	90.00
**Total**	3.96	8.58	5.40	82.06	100.00

Note: Log-linear estimates were derived from a parallel latent class growth analysis (LCGA) model linking hyperactive-inattentive trajectories with social-communication trajectories through jointly fitted multinomial and logit models. This provides identical estimates for β, but trait-specific intercepts. CL = childhood-limited; IM = intermediate; LR = low-risk; PI = persistently impaired; SE = standard error.

a multinomial model (reference: hyperactive-inattentive low-risk group).

b logit model (reference: social-communication low-risk group).

c joint class probabilities were based on estimated posterior probabilities.

**TABLE 1:** Relationships Between Social-Communication and Hyperactive-Inattentive Trait Trajectories

Log-linear Estimates β (SE)				
	Hyperactive-Inattentive Trait Trajectories			
	PI^a^^b^	IM^a^^b^	CL^a^^b^	Intercept^b^
**Social-Communication Trait Trajectories**				
**PI**^a^^b^	5.41 (0.36) (z = 14.87)	3.82 (0.35) (z = 11.06)	2.69 (0.42) (z = 6.38)	−3.89 (0.28)
**Intercept**^a^	−4.74 (0.45)	−2.97 (0.25)	−3.00 (0.20)	

Joint Class Probabilities (%)^c^					
	Hyperactive-Inattentive Trait Trajectories				
	PI	IM	CL	LR	Total
**Social-Communication Trait Trajectories**					
**PI**	3.23	3.90	1.21	1.66	10.00
**LR**	0.71	4.17	4.04	81.08	90.00
**Total**	3.94	8.07	5.25	82.74	100.00

Note: Log-linear estimates were derived from a parallel latent class growth analysis (LCGA) model linking hyperactive-inattentive trajectories with social-communication trajectories through jointly fitted multinomial and logit models. This provides identical estimates for β, but trait-specific intercepts. CL = childhood-limited; IM = intermediate; LR = low-risk; PI = persistently impaired; SE = standard error.

a multinomial model (reference: hyperactive-inattentive low-risk group).

b logit model (reference: social-communication low-risk group).

c joint class probabilities were based on estimated posterior probabilities.

**TABLE 2:** Predictors of Social-Communication and Hyperactive-Inattentive Trait Trajectories

Latent Trajectory Class	Social-Communication^g^		Hyperactive-Inattentive^g^						
PI		PI		IM		CL		Global
OR (95% CI)	*p*	OR (95% CI)	*p*	OR (95% CI)	*p*	OR (95% CI)	*p*	*p*
**Sex**^a^									
Male	**1.88 (1.53–2.32)**	**<.0001**	**3.43 (2.4–4.89)**	**<.0001**	**1.66 (1.3–2.12)**	**<.0001**	**1.82 (1.36–2.46)**	**<0.0001**	**<0.0001**
**Socio-economic position**^b^									
Nonmanual work	1 (0.87–1.6)	.28	1.19 (0.73–1.93)	.48	1.33 (0.93–1.9)	.12	1.24 (0.79–1.95)	0.34	0.32
Materal education									
<O-level^c^	**1.53 (1.08–2.17)**	**.017**	**1.93 (1.14–3.28)**	**.015**	1.42 (0.92–2.2)	.12	1.44 (0.84–2.46)	0.19	**0.038**
O-level^c^	**1.25 (1.01–1.55)**	**.038**	**1.47 (1.05–2.06)**	**.026**	1.26 (0.97–1.63)	.084	1.34 (0.97–1.84)	0.074	**0.023**
**Prenatal maternal risk factors (1st trimester)**^d^									
Alcohol use									
≥1 glass/wk^e^	1.28 (0.95–1.71)	.10	1.31 (0.84–2.05)	.23	1.02 (0.69–1.5)	.94	1.31 (0.84–2.05)	0.24	0.48
<1 glass/wk^e^	1.01 (0.81–1.28)	.91	1.04 (0.73–1.49)	.84	1.09 (0.83–1.42)	.54	1.34 (0.97–1.87)	0.080	0.33
Smoking	**1.45 (1.11–1.9)**	**.0063**	**1.95 (1.34–2.85)**	**.00052**	1.31 (0.94–1.83)	.11	1.33 (0.91–1.96)	0.15	**0.0039**
Marijuana use	1.25 (0.51–3.04)	.62	NE	—	1.72 (0.67–4.39)	.26	2.41 (0.97–5.98)	0.057	0.20
Influenza	1.33 (0.94–1.88)	.11	1.30 (0.75–2.25)	.35	1.28 (0.83–1.96)	.26	1.20 (0.72–2.01)	0.49	0.52
Infections	1.27 (0.99–1.61)	.058	1.27 (0.87–1.85)	.22	1.21 (0.9–1.62)	.21	1.09 (0.76–1.57)	0.64	0.41
**Perinatal risk factors**^d^									
Low birth weight	1.17 (0.68–2.01)	.57	1.53 (0.71–3.3)	0.27	1.09 (0.53–2.24)	.81	1.69 (0.85–3.34)	0.13	0.39
Premature birth	1.19 (0.76–1.88)	.45	1.44 (0.73–2.83)	0.29	1.20 (0.68–2.11)	.54	1.69 (0.94–3.02)	0.081	0.27
Multiparous	0.91 (0.74–1.12)	.38	0.85 (0.61–1.18)	0.33	1.02 (0.79–1.32)	.89	0.99 (0.73–1.35)	0.95	0.83
Maternal age									
<20 years^f^	**2.16 (1.02–4.56)**	**0.043**	**4.32 (1.77–10.54)**	**0.0013**	0.98 (0.24–3.93)	.97	1.54 (0.44–5.37)	0.50	**0.031**
>35 years^f^	0.86 (0.62–1.20)	.37	0.94 (0.57–1.54)	0.79	0.86 (0.58–1.28)	.44	0.76 (0.47–1.26)	0.29	0.63

Note: Findings with *p* ≤ .05 are given in boldface. CL = childhood-limited; IM = intermediate; NE = not estimated; PI = persistently impaired.

a Unadjusted.

b Adjusted for sex.

c Reference: >O-level.

d Adjusted for sex, social class and maternal education.

e Reference: never.

f Reference: 20 to 35 years.

g Reference: low-risk.

**TABLE 3:** Sensitivity Analysis for Exposure to Smoking During Early Pregnancy

Latent Trajectory Class	Social-Communication^c^		Hyperactive-Inattentive^c^						
PI		PI		IM		CL		Global
OR (95% CI)	*p*	OR (95% CI)	*p*	OR (95% CI)	*p*	OR (95% CI)	*p*	*p*
**Additional smoking predictor during early pregnancy**^a^									
Paternal smoking (18 wk)	**1.35 (1.08–1.70)**	**.0094**	**1.53 (1.08–2.15)**	**.016**	1.22 (0.92–1.61)	.16	1.12 (0.81–1.55)	.51	.076
**Adjusted analysis**^b^									
Maternal smoking (first trimester)	1.30 (0.98–1.74)	.072	**1.67 (1.10–2.54)**	**.017**	1.23 (0.86–1.78)	.26	1.33 (0.87–2.03)	.19	.071
Paternal smoking (18 wk)	1.27 (0.99–1.61)	.057	1.32 (0.91–1.91)	.14	1.15 (0.86–1.55)	.35	1.04 (0.73–1.48)	.84	.45

Note: Data with *p* ≤ .05 are in boldface. CL = childhood-limited; IM = intermediate; PI = persistently impaired; wk = weeks of gestation.

a Adjusted for sex, social class, and maternal education.

b In addition to a, paternal smoking (18 wk) and maternal smoking (first trimester) are adjusted for each other.

c Reference: low-risk.

## Supplement 1: Assessment of Maternal Risk Factors During the First Trimester

Information on maternal tobacco use (cigarette smoking) was obtained with the question, “Did you smoke regularly at any of the following times?” and answered by mothers with respect to the first 3 months of the pregnancy. Maternal marijuana use during pregnancy was measured with the question, “How often did you smoke marijuana/grass/cannabis/ganja in the first 3 months of pregnancy?” (Response options: Every day, 2–4 times a week, Once a week, Less than once a week, Not at all). These response options were combined into No (Not at all) and Yes (Remaining options). The frequency of the mother's alcohol consumption was assessed with the question, “How often have you drunk alcoholic drinks?” with respect to the first 3 months of this pregnancy (Response options: Never, <1 glass per week, 1+ glasses per week, 1–2 glasses per day, 3–9 glasses per day, 10+ glasses per day). Because of their lower frequency, response options of 1–2 glasses per day, 3–9 glasses per day, and 10+ glasses per day were combined into 1+ glasses per week.

Information on influenza-like illnesses was measured with the question: “During this pregnancy so far have you had any of the following?” which was answered by mothers with respect to “Influenza in the first 3 months” (Response options: Yes or No). The presence of any infection was indicated if the mothers answered the above question with respect to any urinary infection, influenza, rubella, candida, genital herpes, or other infections during the first 3 months of pregnancy with Yes.

## Supplement 2: Assessment of Additional Smoking Predictors

Mothers' partners were asked, “How many times per day did you smoke at the start of your partner's pregnancy?” (Response options: None, 1–4, 5–9, 10–14, 15–19, 20–24, 25–29, 30+). In addition, mothers were asked about their partner's smoking behaviour at 18 weeks of gestation (“Does your partner smoke?”). Information on paternal smoking from both questions was collapsed into presence or absence of smoking (Response options: Yes and No). Partners who were not the father of the child (or unsure) were excluded from the analysis (0.55%).

## Supplement 3: Assessment of Perinatal Information

Information on parity (number of previous pregnancies resulting in a live birth or still-birth) was collected at 18 weeks of gestation and recoded into “Primiparous” and “Multiparous” mothers. Children's birth weight and gestational age was assessed at birth, and recoded into low birth weight (Yes, <2,500 g; No, ≥2,500 g) and preterm birth (Yes, <37 weeks of gestation; No, ≥37 weeks of gestation). Maternal age at birth was categorized with respect to a teenage pregnancy (<20 years), a normal age range (20–35 years), and an advanced maternal age (>35 years).

## Supplement 4: Assessment of Socio-Economic Position

Data were collected from mothers and their partners at 32 weeks of gestation using questionnaires. Occupational social class was derived as the lower of either maternal or paternal social class, and dichotomized into “Nonmanual” (I, II, III nonmanual) and “Manual” (III manual, IV, V) work.^1^ Information on maternal education was available with respect to O-levels, which are school tests taken approximately at age 16 years in England, and categorized into “Below O-level” (basic school unfinished or some vocational training), “O-level” (minimum school qualifications), and “Above O-level” (higher school-based qualifications).

## Supplement 5: Item Description of the Social Communication Disorder Checklist (SCDC)^2^

The SCDC contains both items that address specific autism spectrum disorder (ASD) symptoms of social impairment and that relate to difficulties with empathy and social insight, as well as other items that are related to the consequences of a lack of social-communicative skill. The former include questions about whether the child is “Aware of other people's feelings”; “Does not realize when others are upset or angry”; “Does not notice the effect of his/her behavior on other members of the family”; “Does not seem to understand social skills”; and “Does not pick up on body language.” The latter items comprise questions such as, “Behaviour often disrupts family life”, “Does not understand how to behave when out”, and “Does not realize if behavior is offensive to other people.”

## Supplement 6: Description of Hyperactive-Inattentive Items on the Strengths and Difficulties Questionnaire (SDQ)^3^

The SDQ assesses hyperactivity-inattention with a five-item subscale (score range 0–10) comprising the following items: “Restless, overactive. Cannot stay still for long” (hyperactive-impulsive), “Constantly fidgeting or squirming” (hyperactive-impulsive), “Easily distracted, concentration wanders” (inattentive), “Thinks things out before acting” (hyperactive-impulsive, reverse coded), and “Sees tasks through to the end. Good attention span” (inattentive, reverse coded). These items are rated as “Not true” (0), “Somewhat true” (1), and “Certainly true” (2) and are added to a summary score. All five items have primary loadings on their intended factor, i.e. hyperactivity-inattention, with minimal cross-loadings on other factors measured by the SDQ.^3,4^
